# Prokaryotic organelle mitochondria drive tumorigenesis: “the original sin”

**DOI:** 10.3389/fonc.2025.1632702

**Published:** 2026-01-02

**Authors:** Chaoyi Wang, Ming Luo, Jinhui Zhou, Qihao Zhang, Xiawei Ji, Jiayao He, Lingfei Wang, Yingpeng Huang, Xiangyang Xue, Fangyan Wang

**Affiliations:** 1 The Second Affiliated Hospital and Yuying Children’s Hospital of Wenzhou Medical University, Wenzhou, China; 2 Zhejiang Key Laboratory of Intelligent Cancer Biomarker Discovery and Translation, First Affiliated Hospital, Wenzhou Medical University, Wenzhou, China; 3 Institute of Inhaled Diagnosis and Therapeutics for Interstitial Lung Disease, Institute of Microbiota and Host Inflammation-related Diseases, School of Basic Medical Science, Wenzhou Medical University, Wenzhou, China

**Keywords:** mitochondria, cancer, endosymbiosis theory, Warburg effect, TCA cycle

## Abstract

Mitochondria preserve bacterial traits because of their endosymbiotic origin, and their alterations in cancer cells reflect these prokaryotic-like traits. One such trait is the Warburg effect, wherein tumor cells rely primarily on aerobic glycolysis instead of oxidative phosphorylation. Cancer cells also exhibit metabolic abnormalities, such as an uncoupled electron transport chain and a truncated tricarboxylic acid (TCA) cycle, potentially generating additional energy. Intermediates from the disrupted TCA cycle can regulate key genes involved in cell differentiation, apoptosis, and tumor suppression while promoting aerobic glycolysis, angiogenesis, and resistance to cell death. Mitochondria-related gene mutations, particularly in D-loop and TCA-related enzymes, have been identified as key drivers of prokaryotic transformation in diverse cancers. Furthermore, the metabolic activity of cancer mitochondria results in the production of essential biosynthetic precursors for nucleotide synthesis and lipid synthesis, supporting tumor growth. Mitochondria also contribute to tumorigenesis by promoting inflammation and iron metabolism disorders. Mitochondrial dysfunctions have raised interest in the use of mitochondria-targeted anticancer strategies as possible cancer treatments, although their clinical application requires further investigation.

## 1 Introduction

Cancer has caused more than ten million mortalities annually worldwide, making it the second leading cause of death. Chemotherapy and radiotherapy still destroy healthy cells, causing toxicity and leading to an immense economic burden on patients. Considering the unsatisfactory cancer survival rate, exploration of epigenetic changes and further investigations into the mechanisms of tumorigenesis are imperative.

Tumor cells are characterized by limitless replication potential, self-sufficient growth signals, resistance to apoptosis, and abnormal cellular energy (1). In addition to the traditional view of cancer as a purely genetic disease, accumulating evidence supports its characterization as a metabolic disorder (2, 3). This paradigm, rooted in the classical Warburg effect, suggests that alterations in cellular energy metabolism are not secondary consequences but primary drivers of tumorigenesis. Consistent with this notion, several hypotheses have proposed nongenetic origins of cancer, emphasizing the pivotal role of sustained metabolic stress, mitochondrial dysfunction, and impaired energy homeostasis in triggering malignant transformation (4). At the ultrastructural level, mitochondria in cancer cells undergo profound morphological alterations, including excessive fission, fragmentation, and disruption of crista organization (5). These changes correlate closely with functional impairments, such as loss of membrane potential, defective oxidative phosphorylation, and increased reactive oxygen species production (5). Collectively, these structural and functional alterations reinforce a vicious cycle of metabolic reprogramming that fuels tumor growth, survival, and adaptation (6).

Interestingly, within eukaryotes, mitochondria are unique organelles that resemble those of prokaryotic cells. The concept that mitochondria originate from free-living bacteria integrated into a primitive eukaryotic ancestor is rooted in endosymbiotic theory, which was first formally proposed and developed by Lynn Margulis (7, 8). According to this theory, eukaryotes are derived from a primitive cell that produces energy via glycolysis and accidentally engulfs oxygen-consuming bacteria, which evolve into mitochondria (9). Trigos et al. proposed that during tumorigenesis, gene expression reverts toward an evolutionarily ancient state, with tumors preferentially upregulating genes of unicellular origin while downregulating those associated with multicellular functions. When mitochondrial structure or function becomes disrupted, cells can shift toward a more primitive, glycolysis-based energy state, similar to that of their bacterial predecessors (10, 11). This regression supports uncontrolled growth and resistance to stress, linking mitochondrial dysfunction to the metabolic flexibility and malignant behavior of cancer cells.

In this review, we summarize the current understanding of mitochondrial metabolic reprogramming and the impact of mitochondrial gene mutations and structural changes on cancer progression. We also discuss emerging therapeutic strategies targeting mitochondrial dysfunction, highlighting the promise and challenges of these approaches.

## 2 The physiological function of mitochondria, endosymbiotic prokaryotic cells

The prevailing hypothesis on the origin of mitochondria is the endosymbiosis theory, which proposes that the origin of eukaryotic cells can be traced to ancestral nucleated cells. The ancestral cells engulf aerobic gram-negative bacteria to establish a symbiotic relationship wherein the bacteria provide energy to the host cell through aerobic respiration. The symbiosis enables a portion of bacterial DNA to integrate into the host genome, thereby achieving intergenerational genetic transfer, allowing bacteria to gradually evolve into mitochondria (12). Importantly, mitochondria couple glycolysis in the host cell cytoplasm with the TCA cycle and oxidative phosphorylation (OXPHOS), generating a substantial amount of ATP, which is essential for host survival and competitive advantage. Furthermore, mitochondria play an important role in one-carbon metabolism, which begins in mitochondria, where serine hydroxymethyltransferase (mSHMT) converts serine to glycine, generating 5,10-methylene-tetrahydrofolate, a key one-carbon donor in the folate cycle. It transfers one-carbon units to the precursor molecules of purines and pyrimidines, supporting their synthesis (13). However, in the transition to a malignant state, mitochondria undergo significant ultrastructural modifications, supporting their dysfunction. The delicate balance of mitochondrial dynamics is lost, typically favoring excessive fission and leading to a fragmented mitochondrial network (14). Concurrently, the inner mitochondrial membrane undergoes remodeling, with disorganization of the cristae structure. As demonstrated in studies, these ultrastructural defects are mechanistically linked to the functional impairments observed in cancer cells, such as electron transport chain inefficiency, compromised oxidative phosphorylation, and aberrant apoptotic signaling (15). This structure–function relationship provides a physical basis for the metabolic disorders that characterize cancer mitochondria. Disruptions in mitochondrial function, along with the reemergence of ancestral prokaryotic features, may act as driving factors in cancer development, which we discuss in the following section.

## 3 Metabolic disorders in cancer mitochondria

### 3.1 Mitochondria-driven hypermetabolic state in tumor cells

#### 3.1.1 The Warburg effect

During tumorigenesis, the Warburg effect is postulated to be a key driver (16, 17). The Warburg effect is common in tumors, in which tumor cells undergo aerobic glycolysis with lactate secretion rather than OXPHOS for energy production, even in the presence of oxygen (18). The occurrence of the Warburg effect involves multiple pathways. For instance, the hypoxia-inducible factor-1α (HIF-1α) and phosphoinositide 3-kinase (PI3K)/protein kinase B (Akt) signaling pathways induce key glycolytic enzymes, such as pyruvate kinase M2 (PKM2), hexokinase 2 (HK2), and lactate dehydrogenase A (LDHA), to strengthen the Warburg effect over time (19–21).

PKM2 is a widely detected isotype of pyruvate kinase in cancer patients who transfers phosphate from phosphoenolpyruvate (PEP) to adenosine diphosphate (ADP) to complete glycolysis. Unlike its splice variant isotype PKM1, PKM2 displays low pyruvate kinase (PK) activity but diverts glycolytic substrates into alternative biosynthetic pathways and reduces nicotinamide adenine dinucleotide phosphate (NADPH)-generating pathways. Additionally, the hydroxylation of PKM2 promotes binding to HIF-α and further transactivation of HIF-targeted genes to adapt to hypoxia (22–24). Although it may seem counterintuitive for cancer to exhibit decreased PKM2 activity in the context of the Warburg effect, it has been suggested that this reduction in PKM2 activity actually enhances the flow of glycolytic intermediates into biosynthetic pathways. This pathway includes the pentose phosphate pathway for nucleotide synthesis, as well as pathways for serine biosynthesis (25). This finding suggests that the Warburg effect is more likely to function as a hub for producing metabolites that tumor cells require, rather than primarily generating energy.

Overall, the Warburg effect represents a critical metabolic adaptation that provides a rapid but inefficient source of ATP. However, accumulating evidence indicates that mitochondrial respiration remains active in many highly glycolytic tumors (26), challenging the notion of a complete metabolic shift from oxidative phosphorylation to glycolysis. Therefore, we propose that tumor cells may integrate both glycolytic and mitochondrial pathways to meet the high demands of proliferation and survival.

#### 3.1.2 Uncoupled electron transport complex

Although the Warburg effect is a dominant metabolic hallmark of cancer, tumor cells do not uniformly suppress oxidative phosphorylation (OXPHOS). Several studies have shown elevated activities of electron transport chain (ETC) complexes I, II, and IV in breast cancer, classical Hodgkin lymphoma, and diffuse large B-cell lymphoma, indicating that mitochondrial respiration remains functionally active in many malignancies (27, 28). This evidence suggests that, rather than abandoning OXPHOS, tumor cells retain or even increase ETC activity to complement glycolysis and sustain energy homeostasis. Under hypoxic or nutrient-limited conditions, however, electron donors such as NADH and FADH_2_ may accumulate due to restricted oxygen availability, leading to electron leakage and redox imbalance. To circumvent this, cancer cells may develop mechanisms analogous to those of prokaryotes, employing alternative electron acceptors or partial respiratory processes to maintain metabolic flux and redox stability (29–32).

Consistent with the activity of ETC proteins, the TCA cycle is hyperactivated in many tumors. Most TCA enzymes are upregulated, with the exception of succinate dehydrogenase (SDH), leading to succinate accumulation and substrate-level phosphorylation via succinyl-CoA synthetase to generate GTP (33, 34). These findings indicate that tumor mitochondria exploit both uncoupled ETC flux and a modified TCA cycle to sustain ATP production and biosynthetic demands, even in the face of impaired oxidative phosphorylation. Moreover, the ETC function can be fine-tuned through the selective expression of subunit isoforms. Hypoxia induces COX IV-2, an isoform with lower catalytic efficiency than COX IV-1 does, thereby reducing oxygen consumption and reinforcing glycolytic reliance (35). Conversely, high glucose suppresses COX I and COX IV-1 expression, further shifting metabolism toward aerobic glycolysis (35). Alterations in complex I subunits (NDUFS3, NDUFA13) and complex III components (cytochrome b, Rieske iron–sulfur protein) increase reactive oxygen species (ROS) production, which in turn activates oncogenic signaling pathways (36–38).

Taken together, these observations demonstrate that cancer cells do not simply switch off mitochondrial respiration but rather reprogram ETC and TCA dynamics. However, the literature remains inconsistent regarding whether ETC upregulation supports or opposes tumor growth, as both hyperactive and repressed ETC states have been reported across cancer types. This discrepancy may reflect differences in tumor oxygenation, nutrient availability, and oncogenic signaling.

### 3.2 Truncated tricarboxylic acid cycle

Rather than functioning solely as an energy-generating pathway, the TCA cycle in tumor cells is frequently reprogrammed into a truncated form that simultaneously supports biosynthesis and redox homeostasis. In many cancers, the preferential export of citrate from mitochondria fuels the synthesis of amino acids, fatty acids, and nucleotides essential for rapid proliferation while limiting the complete oxidation of carbon substrates (39–42). Glutamine metabolism plays a central role in this truncated TCA cycle. In tumor mitochondria, the upregulation of glutamate dehydrogenase 1 (GDH1) in glutamine metabolism promotes an increase in the levels of α-ketoglutarate (α-KG) and the subsequent intermediate metabolite fumarate, which activates and binds to the ROS scavenging enzyme glutathione peroxidase 1 (GPX1) to protect against oxidative damage (43). The levels of the intermediates malic acid and isocitrate increase and promote the activity of their corresponding mitochondrial enzymes, leading to NADPH generation (44). In parallel, the malic and isocitrate dehydrogenase reactions contribute additional NADPH, reinforcing redox defense while maintaining anabolic fluxes (45). Consistent with this metabolic remodeling, breast cancer samples with increased malignancy present elevated levels of malic and fumaric acids, supporting the existence of a truncated and biosynthetically active TCA cycle (46). However, this adaptive reprogramming can create vulnerabilities. In IDH1/2-mutated tumors such as gliomas, aberrant conversion of α-KG to the oncometabolite 2-hydroxyglutarate consumes NADPH and disrupts the cellular redox balance, increasing the susceptibility of cells to oxidative damage (47, 48).

In cancer, mutations in TCA cycle enzymes, including succinate dehydrogenase (SDH), fumarate hydratase (FH), and isocitrate dehydrogenase (IDH), are unifying features of the accumulated TCA cycle intermediates (49). SDH and FH loss-of-function mutations cause the accumulation of succinate and fumarate, respectively, which inhibit α-KG–dependent dioxygenases, stabilize HIF1α, and modify proteins such as KEAP1, thereby promoting pseudohypoxia, Nrf2-driven antioxidant responses, and transcriptional rewiring that favor tumorigenesis (49–51). SDH- and FH-deficient tumors also display a wide range of clinical behaviors. SDH mutations are frequently associated with hereditary paragangliomas and gastrointestinal stromal tumors that are generally benign. In contrast, the same defect in renal tissue can lead to highly aggressive renal cell carcinomas with early metastasis (52–54). This contrast suggests that the biological impact of oncometabolite accumulation strongly depends on the tissue context, cooperating nuclear mutations, and microenvironmental conditions. IDH1/2 mutants consume NADPH to produce D-2-HG, which similarly inhibits α-KG–dependent chromatin and DNA demethylases, driving a hypermethylation phenotype that can initiate tumorigenesis, particularly in gliomas and hematologic malignancies (55, 56).

**Figure 1:**
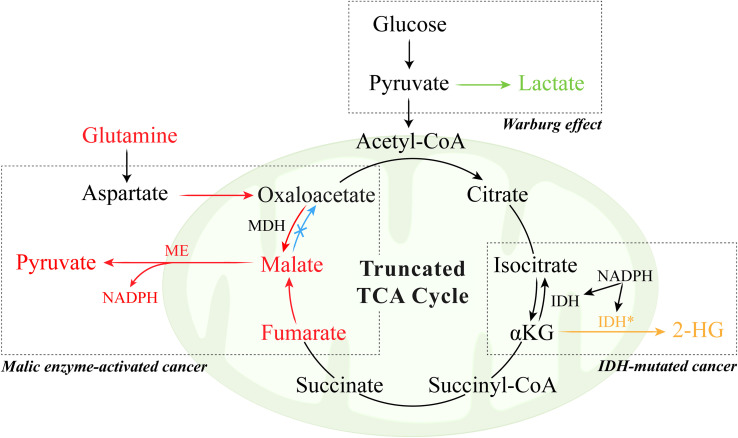
Warburg effect and truncated TCA cycle in tumor.

Overall, truncation of the TCA cycle represents a dual adaptive mechanism that facilitates anabolic growth and maintains redox stability under fluctuating oxygen conditions. However, the relative contribution of each function likely depends on the tumor type, microenvironmental oxygen level, and oncogenic context. In gliomas, for example, IDH mutations may function as early drivers but later become passenger alterations as more aggressive mutations emerge (57). Future studies combining isotope tracing and redox flux analysis are needed to clarify how TCA flexibility balances bioenergetic demands with oxidative stress tolerance in cancer metabolism (**Figure 1**).

### 3.3 MtDNA mutation and mitochondrial transfer

Human mitochondrial DNA (mtDNA) is a 16.5 kb circular, double-stranded molecule that is independent of the nuclear genome (58). Lacking introns and efficient repair mechanisms and being exposed to high ROS levels from the ETC, mtDNA has a mutation rate over 10 times higher than that of nuclear DNA (59). Emerging evidence links mtDNA variation to tumorigenesis. Point mutations, insertions, and deletions have been identified in various tumor types, with base substitutions being the most common (59–62). These mutations can exhibit chain-biased replication, particularly C>T and A>G on the mitochondrial heavy chain, primarily due to oxidative inactivation of DNA polymerase-γ (63).

Emerging evidence suggests that mtDNA mutations can contribute to tumor progression. A genome-wide association study revealed that susceptibility to perimenopausal breast cancer significantly increases when a patient has a maternal grandmother with the same type of breast cancer, suggesting that mtDNA mutations may function as a predisposing factor for the specific subtype of breast cancer (64). Tan et al. examined mutations in the entire mitochondrial genome across 19 pairs of normal and tumor breast tissues sourced from the same patients, revealing that somatic mutations were present in 74% of cases. The D-loop is a 1,124-bp noncoding region of mitochondrial DNA that contains crucial transcription and replication elements. Mutations in the D-loop, which houses the initiation site for replication and transcriptional promoters, can alter the mtDNA copy number (65). A total of 81.5% of these mutations were localized to the D-loop region, while mutations were also observed in the 16S rRNA, ND2, and ATPase 6 genes (62). Barekati et al. reported that in the tissues of breast cancer patients lacking TP53 mutations, the mutation rate in the D-loop region of normal somatic cells was 36.36%, whereas that of germline cells reached 90.91%, indicating that mitochondrial mutations could originate from maternal genes (66).

In addition to genetic alterations, mitochondria can move between cells through tunneling nanotubes, extracellular vesicles, or direct cell–cell contact, thereby reprogramming the metabolic and functional state of recipient cells (67). Such transfer has been observed between stromal and cancer cells, neurons and glioma cells, and even between immune and tumor cells (68–70). In the tumor microenvironment, functional mitochondria supplied by stromal or mesenchymal cells can restore oxidative phosphorylation in cancer cells, increase their invasive potential, and promote therapy resistance (71). Conversely, tumor cells can donate damaged or “tolerant” mitochondria to infiltrating T cells, impairing T-cell metabolism and leading to functional exhaustion, which contributes to immune evasion and resistance to immunotherapy (72). This cross-cellular organelle transfer challenges the traditional view of mitochondria and reflects certain prokaryotic behaviors, such as metabolic cooperation at the community level. Mitochondrial transfer represents a new layer of tumor plasticity that operates in parallel with genetic and metabolic reprogramming. However, the field remains in its early stages, with most mechanistic insights derived from *in vitro* or animal models, and quantitative *in vivo* evidence in human tissues remains limited. Future studies combining *in situ* mitochondrial labeling, spatial metabolomics, and lineage tracing may clarify the prevalence and consequences of mitochondrial transfer in human cancers.

Collectively, mtDNA mutations and mitochondrial transfer highlight the dynamic and semiautonomous nature of mitochondria in tumor evolution. Mitochondria can transmit not only genetic information vertically but also metabolic capacity horizontally, thereby reshaping cancer metabolism and the therapeutic response.

### 3.4 Promotion of the synthesis of substances required for tumor cell proliferation

#### 3.4.1 Fatty acid synthesis

During tumorigenesis, glutamine metabolism is significantly active in lipid biosynthesis (73). Glutamine is first converted into glutamate by glutaminase (GLS) 1 in the mitochondria and is further catalyzed into α-KG under the action of glutamate dehydrogenase 1 (GLUD1) (74). When α-KG enters the truncated TCA cycle, it is oxidized into succinate, fumarate, and malic acid in turn. Malic acid is then shuttled to the cytoplasm and decarboxylated into pyruvate, accompanied by substantial NADPH production (75, 76). However, when tumor proliferation exceeds the oxygen supply, cells encounter anaerobic conditions and employ an alternative pathway to produce NADPH for redox balance and *de novo* lipid synthesis. This process begins with the reduction of glutamine-derived α-KG to isocitrate by NADPH-dependent IDH, followed by the generation of citrate. Subsequently, ATP citrate lyase (ACLY) catalyzes the conversion of citrate to acetyl-CoA and oxaloacetate. Acetyl-CoA is a crucial precursor for subsequent fatty acid synthesis (77, 78). It has been estimated that 80% of acetyl-CoA is involved in fatty acid synthesis under hypoxia, whereas 10% to 25% is involved in fatty acid synthesis under normal culture conditions, which suggests that an efficient reductive carboxylation reaction helps tumor cells survive hypoxia (79).

#### 3.4.2 Nucleic acid synthesis

**Figure 2:**
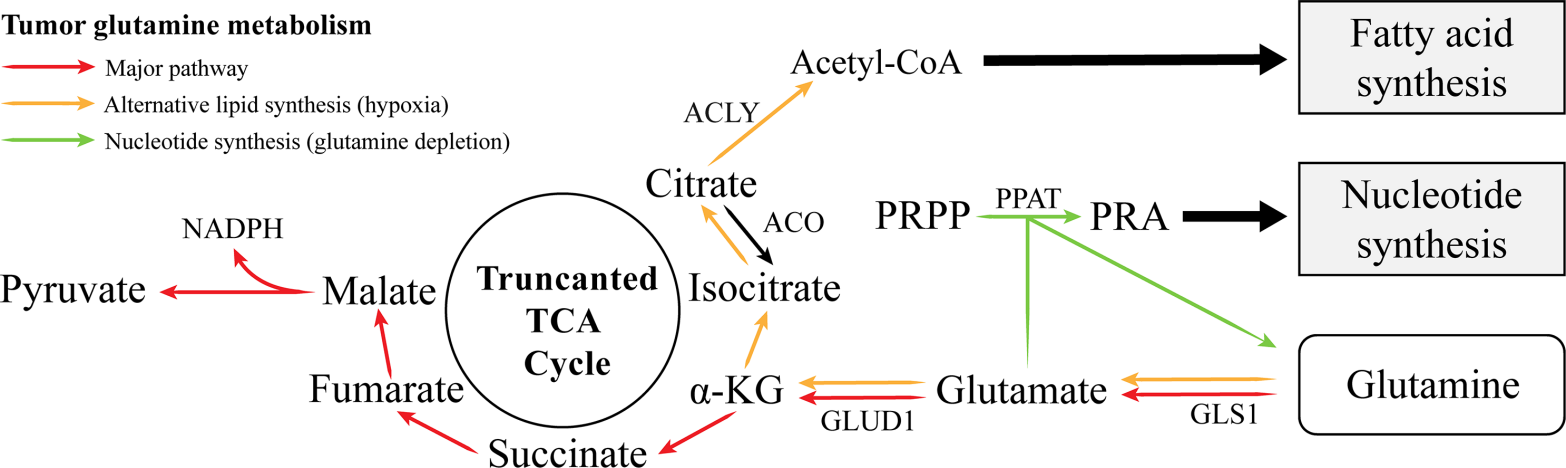
Abnormal glutamine metabolism supports lipid and nucleotide synthesis in cancer.

In addition to supplying carbon for the biosynthetic pathway, glutamine also functions as an essential nitrogen donor in the biosynthesis of amino acids and nucleotides (80). Glutamate can undergo transamination with oxaloacetate via transaminase to produce aspartate in the purine nucleotide cycle. In purine nucleotide biosynthesis, the amide nitrogen from glutamine is transferred to phosphoribosyl pyrophosphate (PRPP) by phosphoribosyl amidotransferase (PPAT) to form phosphoribosylamine (PRA), the first purine nucleotide precursor. Aspartate also contributes to pyrimidine synthesis (80). Therefore, since glutamine metabolism plays a vital role in biosynthesis and its inhibition limits tumor cell progression, some scholars have postulated that glutamine deprivation causes cell cycle arrest in the S phase in certain tumor types (81). Moreover, in the core region of solid tumors, tumor cells are usually glutamine deficient. To restore nitrogen metabolism under glutamine-restricted conditions, tumor cells upregulate PPAT expression and inhibit mitochondrial GLS1. In this case, lactate replaces glutamine as a carbon source in the truncated TCA cycle (82, 83). In a recent meta-analysis that included approximately 11,000 patients, the transamination of glutamine played a crucial role in the malignant progression of tumors, and its enzyme PPAT may be a prospective prognostic marker (84). Thus, the role of mitochondria is closely related to the function of the truncated TCA cycle in tumor cells. Mitochondria utilize glutamine metabolism to regulate intermediate metabolites of the TCA cycle, which maintains the biosynthetic precursor library and completes the biosynthesis required for cell proliferation (**Figure 2**).

## 4 Cancer-promoting cellular behavior induced by mitochondria

### 4.1 Intermediate metabolites in the TCA cycle modulate the host gene expression profile

#### 4.1.1 Antagonists for epigenetic modification enzymes

Recent studies have shown that intermediate metabolites in the TCA cycle, such as succinate, fumarate, and 2-HG, can alter gene expression profiles. These metabolites are structurally similar to α-KG and compete with α-KG for binding to a variety of α-KG-dependent dioxygenases, such as JmjC domain-containing histone demethylases (KDMs), prolyl hydroxylases (PHDs), collagen prolyl-4-hydroxylase (C-P4H), and the ten-eleven translocation (TET) family of 5-methylcytosine (5mC) hydroxylases (85). Among them, the inhibitory effect of 2-HG was the weakest, whereas that of fumarate was the strongest (86). This inhibition contributes to pseudohypoxia and significantly impacts epigenetic and other biological processes.

In the cytoplasm, the inhibition of PHDs by tumor metabolites activates and stabilizes HIF-1α (87). Even under aerobic conditions, HIF-1α can be stabilized, resulting in pseudohypoxia, which plays an important role in the processes of angiogenesis, metabolism, proliferation, and metastasis to promote tumorigenesis (88, 89). Consistent with this, HIF-1α is usually not expressed in most normal human tissues but is expressed in 53% of malignant tumors (90). Its accumulation increases the expression of glucose transporters 1 and 3 to promote the entry of glucose into cells (91, 92), and its activation increases the expression of glycolytic enzymes such as hexokinase (HK)1 and HK2 and promotes the activity of LDHA to increase glycolysis (93). This continuous aerobic glycolysis is consistent with the Warburg effect and benefits tumor growth. In addition, its expression level has been proven to be positively correlated with malignancy, invasion, and migration, and contributes to higher adverse event rates (94). Further studies have shown that HIF-1α in hepatocellular carcinoma activates the Akt/mTOR/STAT3 signaling pathway through IL-8 (95), and HIF-1α in colorectal cancer reduces E-cadherin and increases vimentin through increased activity of zinc finger E-box 1 (ZEB1), thus promoting epithelial–mesenchymal transition, tumor cell migration, and invasion (96).

Tumor metabolites can inhibit Jumonji C domain–containing histone lysine demethylases (KDMs) and the ten-eleven translocation (TET) family of 5mC hydroxylases, thereby preventing histone demethylation and impairing DNA demethylation. As a result, the inhibition of these enzymes leads to a genome-wide hypermethylation phenotype in both histones and DNA (97–99). Promoters are often located within CpG islands, regions of the genome rich in cytosine and guanine nucleotides. Hypermethylation of these CpG islands typically silences tumor suppressor genes (100). Additionally, increased DNA methylation of specific genes, such as miR-200, can suppress gene expression and promote epithelial-to-mesenchymal transition, potentially contributing to tumor cell invasion and metastasis (101).

#### 4.1.2 Substrates for epigenetic modification

Some metabolites in the TCA cycle, such as acetyl-CoA and succinyl-CoA, can be used as substrates for epigenetic modifications and dramatically influence the host gene expression profile.

Acetyl-CoA serves as a substrate for lysine acetyltransferases, enzymes that transfer the acetyl group to lysine residues on histones and other proteins, thereby acetylating them (102, 103). When glucose is sufficient, the concentration of acetyl-CoA increases. As a signal of nutritional adequacy, acetyl-CoA induces histone acetylation, relaxes the chromatin structure, facilitates transcription factor binding, activates the expression of genes involved in proliferation, and promotes cell growth and proliferation (103, 104). After glucose deprivation, acetyl-CoA synthetase 2 (ACSS2) enters the nucleus, binds to the transcription factor EB, and converts the acetate produced by the deacetylation of histone or other nucleoproteins into acetyl-CoA, which is used for histone acetylation in the promoter region of lysosome and autophagosome genes, thus enhancing autophagy and lysosome biosynthesis to maintain intracellular homeostasis during tumor growth (105). Moreover, ACSS2 also upregulates the expression of the autophagy-related factor LAMP1 to promote autophagy, migration, and invasion in tumor cells (106). The downregulation of acyl CoA thioesterase 12 (ACOT12) contributes to the accumulation of acetyl-CoA, which leads to increased histone acetylation and activation of the TWIST2 gene, thereby promoting epithelial–mesenchymal transition and tumor metastasis (107). In addition, lysine acetylation of nonhistone proteins plays an important role in tumor growth and proliferation. For example, acetylation of phosphoglycerate kinase 1 at lysine residue 323 enhances its activity and promotes glycolysis (108). Acetylation of PKM2 at lysine residue 305 reduces its activity and leads to the accumulation of glycolytic intermediates that support cell growth (109). Mitochondrial acetyl-CoA acetyltransferase can specifically acetylate pyruvate dehydrogenase, reducing its activity and promoting the Warburg effect and tumor growth (110). The acetylation of ATP citrate lyase at lysine residues 540, 546, and 554 promotes *de novo* lipogenesis (111). These changes in cell metabolism significantly promote cell proliferation and tumor growth.

As mentioned above, mutations in genes encoding SDH, FH, and IDH contribute to the accumulation of succinate, fumarate, and 2-HG. Still, some studies have shown that their upstream product, succinyl-CoA, is also elevated, resulting in the succinylation of lysine (112). This succinylation of lysine first occurs on mitochondrial proteins and promotes the expression of the antiapoptotic gene Bcl-2 on the mitochondrial membrane, increasing lactate production and the pyruvate-to-citrate ratio (112). Further investigations revealed that the α-KG dehydrogenase (α-KGDH) complex can also enter the nucleus and bind to lysine acetyltransferase 2A (KAT2A). The α-KGDH complex subsequently catalyzes the local synthesis of succinyl-CoA, which competes with acetyl-CoA, whereas KAT2A succinylates histone H3 on lysine 79 and regulates important genes such as p85α, c-Jun, and DNA-PKcs. These modifications promote tumor growth (113).

### 4.2 MtDNA release promotes cancer-associated inflammation

Mitochondrial dysfunction and inflammation form a critical axis in cancer development. Recent studies have shown that the release of mtDNA in the pro-cancer microenvironment acts as a crucial bridge in mitochondria-induced inflammation (114). Unlike nuclear DNA, mtDNA has immunostimulatory properties due to its lack of CpG methylation and its bacterial-like circular structure (114). Once released, cytosolic mtDNA is recognized by cyclic GMP–AMP synthase (cGAS), which catalyzes the synthesis of cyclic GMP–AMP (cGAMP) to activate the adaptor STING located on the endoplasmic reticulum or inner membrane system (115, 116). STING then recruits TBK1 and promotes IRF3 phosphorylation, leading to type I interferon and proinflammatory gene expression through IRF3 and NF-κB signaling (114). Moreover, mtDNA internalized into endolysosomal compartments can activate Toll-like receptor 9 (TLR9), and oxidized mtDNA serves as a potent activator of the NLRP3 inflammasome, inducing IL-1β and IL-18 maturation (117). The mechanisms underlying mtDNA release include mitochondrial outer membrane permeabilization (MOMP) through Bax/Bak pores, opening of the mitochondrial permeability transition pore (MPTP), voltage-dependent anion channel (VDAC) oligomerization, and mitochondrial-derived vesicle (MDV) formation, which are often exacerbated by defective mitophagy (118–121). Dysfunction of the inner membrane GTPase optic atrophy protein 1 (OPA1) in tumors destabilizes cristae structure and increases mitochondrial susceptibility to outer membrane permeabilization, facilitating mtDNA escape into the cytosol (122).

MtDNA release has been shown to activate the NLRP3 inflammasome, triggering inflammatory responses (116). Mitochondrial ATP binds to the NACHT domain of NLRP3, supplying energy for inflammasome assembly and activation (123). Subsequently, Caspase-1 processes the proinflammatory cytokines IL-1β and IL-18, converting them into their active forms and cleaving gasdermin proteins to promote pyroptosis (124). Thus, high levels of inflammatory cytokines, such as IL-1β, are common in various cancer types and recognized as potential cancer biomarkers (125). In addition to direct inflammatory activation, these cytokines can increase vascular permeability and attract inflammatory cells, such as macrophages, neutrophils, and myeloid-derived suppressor cells (MDSCs), to the tumor microenvironment (126). Notably, the shift of mitochondria toward proinflammatory signaling in cancer cells provides opportunities for therapies targeting this cancer-promoting microenvironment. Pharmacological inhibition of VDACs can reduce mtDNA release, decrease extracellular mtDNA levels, reverse PMN-MDSC-driven immunosuppression, and enhance chemotherapy efficacy in prostate cancer mouse models (127). An IL-1 receptor antagonist (IL-1RA) has been shown to block MDSC recruitment and inhibit tumor progression in gastric cancer models (128).

### 4.3 Disrupted mitochondrial iron metabolism promotes tumor progression

As hubs of iron metabolism, mitochondria require sufficient iron to maintain normal physiological functions, a trait linked to their prokaryotic origins. They utilize cellular iron to synthesize essential cofactors, including heme and iron–sulfur clusters (129). Research has shown that abnormal iron homeostasis in tumor cells is manifested mainly by increased expression of ferritin, transferrin, transferrin receptor, and hepcidin, leading to increased iron absorption and storage and ultimately causing iron overload in tumor cells (130–133). Iron usually exists in the mitochondria as iron–sulfur clusters in typical proteins, one of the most abundant and functionally pliable redox proteins found in all living organisms, including complexes I, II, and III (134). In tumor cells, increased ETC activity may be due to iron overload (135, 136).

Ni et al. reported that mitochondrial iron accumulation is greater than that in the cytoplasm in tumor cells, which suggests the critical role of mitochondrial iron homeostasis in tumor development (137). When iron is overloaded in the mitochondria, heme synthesis is dramatically elevated, and excessive production contributes to p53 protein degradation via the ubiquitin production pathway, partly explaining the decrease in p53 protein in tumors (138). As an iron chaperone essential for cell survival, frataxin plays a key role in modulating mitochondrial iron homeostasis, including iron storage and detoxification, the regulation of iron metabolism, and protection against proapoptotic stimuli. Owing to iron overload, decreased expression of frataxin hinders the synthesis of the iron-dependent enzyme cytochrome C in mitochondria, which primarily inhibits caspase-3-dependent apoptosis and rapid programmed cell death (139–141). Additionally, the p53 protein can bind to the promoter region of frataxin, mediate its transcription, and control mitochondrial iron metabolism, glycolysis, and apoptosis (142, 143). The complex formed by p53 and the BclXL/Bcl-2 proteins destabilizes the outer mitochondrial membrane and allows the release of cytochrome C to initiate apoptosis (144). In tumor cells, TP53 gene mutations contribute to a significant decrease in p53 expression, which reduces the TP53-induced expression of glycolysis and apoptosis regulator (TIGAR), thereby enhancing the Warburg effect (143, 145). Moreover, excess iron catalyzes the Fenton reaction, generating reactive oxygen species (ROS), which inhibit the prolyl hydroxylases (PHDs) responsible for HIF-1α degradation. As a result, stabilized HIF-1α translocates to the nucleus and upregulates the expression of glycolytic enzymes and glucose transporters, enhancing glycolytic flux even under normoxic conditions (146).

Notably, iron–sulfur clusters play a critical role in tumorigenesis (147). Iron–sulfur clusters in the mitochondrial respiratory chain are found in NADH-ubiquinone oxidoreductase (Complex I), Rieske iron–sulfur protein (RISP), and subunits of SDH (148). Complex I contains eight iron–sulfur clusters, a flavin mononucleotide, and ubiquinone, and its activity affects the cellular NAD pool and the NAD/NADH ratio, which are important for mitochondrial malate dehydrogenase (149, 150). Previous research has suggested that complex I inhibitors, such as metformin, rotenone, and IACS-010759, disrupt the NAD/NADH balance, leading to a decrease in available electron acceptors (151–153). As a result, aspartate synthesis is limited, which interferes with purine and pyrimidine biosynthesis and inhibits cell proliferation (154). Moreover, SDH contains three distinct iron–sulfur clusters. Its abnormal expression is linked to an increased risk of pheochromocytomas and paragangliomas (155, 156).

**Figure 3:**
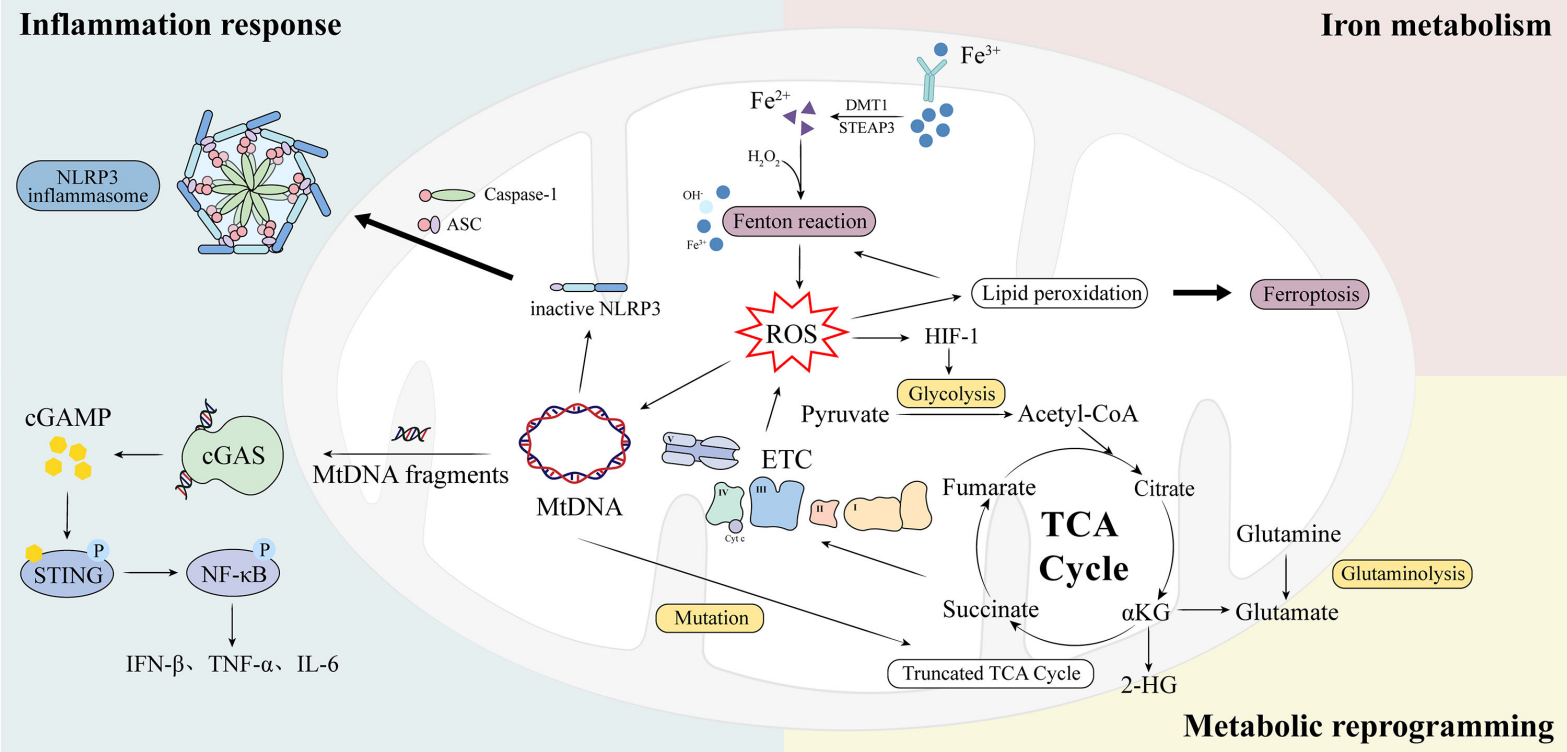
Overview of the interconnections among prokaryotic-like transition of mitochondria in cancer.

Collectively, during tumorigenesis, mitochondria may develop interrelated behaviors that promote one another, involving reprogrammed metabolism, mitochondria-induced inflammation, and disruptions in iron metabolism (**Figure 3**).

## 5 Mitochondria-targeted anticancer strategies

Cancer cells can reprogram mitochondrial function to sustain bioenergetic and biosynthetic demands, evade apoptosis, and adapt to microenvironmental stress. Consequently, targeting mitochondrial pathways has emerged as a promising strategy to disrupt tumor metabolism and survival. Recent studies have identified multiple mitochondrial vulnerabilities, including OXPHOS, mitochondrial translation, apoptosis regulation, redox homeostasis, and organelle dynamics. The following sections summarize key therapeutic approaches that interfere with mitochondrial function and signaling, highlighting their mechanisms of action, preclinical efficacy, and translational challenges (**Table 1**).

**Table 1:** Mitochondria-targeted anticancer strategies.

Strategy	Mechanistic target/pathway	Representative agents	Preclinical/clinical stage	Limitations/challenges
Metabolic inhibitors	OXPHOS complexes (Complex I–V); TCA cycle enzymes (PDH, KGDH)	IACS-010759 (Complex I inhibitor); CPI-613/devimistat (PDH/KGDH modulator)	Most agents are preclinical to early clinical	Dose-limiting toxicity; metabolic compensation; limited efficacy.
5.2 Mitochondrial translation inhibitors	Mitochondrial ribosomes	Tigecycline, Doxycycline	Mostly preclinical/repurposing clinical studies for cancer and mitochondrial disease models.	Affects normal stem & immune cells; systemic toxicity.
5.3 Modulators of mitochondrial apoptosis	BCL-2 family proteins; MOMP regulation	Venetoclax (BCL-2 inhibitor), Navitoclax (BCL-2/BCL-xL dual inhibitor); modulators of VDACs & mPTP	Some agents approved (venetoclax), others in late/early clinical trials or preclinical.	Resistance; on-target toxicity; limited efficacy.
5.4 Redox-directed therapies	Mitochondrial ROS & antioxidant systems	Elesclomol (Cu ionophore), Piperlongumine, RSL3 (GPX4 inhibitor), FSP1 inhibitors	pro-oxidants mostly in preclinical/early clinical oncology trials; mitochondrial antioxidants/peptides in preclinical and clinical trials for mitochondrial/myopathies and cardiac disease.	Narrow therapeutic window; systemic oxidative stress; hypoxia-induced ROS adaptation.
5.5 Modulators of mitochondrial biogenesis & dynamics	Mitochondrial fission, fusion, replication	mdivi-1 (DRP1 inhibitor); PGC-1α, TFAM, MFN2 modulators	Mostly preclinical or early clinical.	Context-dependent effects; Off-target impact on normal tissues.

### 5.1 Metabolic inhibitors

Several small molecules that target OXPHOS and the TCA cycle have shown therapeutic potential. IACS-010759 is a potent inhibitor of mitochondrial complex I. The drug blocks electron transfer by binding to complex I at the quinone/ND1-associated site, thereby suppressing OXPHOS and electron transport (157). Inhibition of complex I by IACS-010759 leads to reduced cellular ATP production and decreased aspartate and nucleotide biosynthesis (157, 158). Consequently, the compound exhibits significant antitumor activity in preclinical models of leukemia and glioma (159). However, clinical development was discontinued due to a narrow therapeutic window and dose-limiting toxicities (159). CPI-613 (devimistat) targets the lipoate-dependent enzymes pyruvate dehydrogenase (PDH) and α-ketoglutarate dehydrogenase (KGDH), thereby inhibiting TCA cycle flux, reducing NADH production, and disrupting the mitochondrial redox balance (160). In preclinical models of acute myeloid leukemia (AML) and pancreatic and lung cancers, treatment with CPI-613 leads to decreased ATP levels, accumulation of ROS, and induction of apoptosis (161). However, subsequent clinical trials demonstrated limited efficacy (162).

In clinical practice, tumor cells can compensate for mitochondrial blockade by enhancing glycolysis or fatty acid oxidation as a resistance mechanism. Combination strategies, such as pairing OXPHOS inhibitors with glycolytic blockers, antiangiogenic agents, or immune checkpoint therapy, are being explored to overcome compensation. Moreover, systemic inhibition of mitochondrial respiration risks toxicity in high-energy tissues such as the heart and brain. Therefore, identifying tumor subtypes that rely on OXPHOS is essential for clinical translation.

### 5.2 Mitochondrial translation inhibitors

Given their bacterial ancestry, mitochondria retain a ribosomal system similar to prokaryotes. Except for complex II, all OXPHOS complexes contain subunits encoded by mtDNA, thus relying on mitochondrial ribosomal translation (163). Several antibiotics, such as tigecycline, doxycycline, and azithromycin, selectively inhibit mitochondrial ribosomes and thereby suppress the synthesis of mtDNA-encoded OXPHOS subunits. Tigecycline markedly reduces the activity of mitochondrial respiratory chain complexes I and IV by inhibiting mitochondrial translation, leading to a decreased oxygen consumption rate (OCR), energy depletion, and oxidative stress (164). By targeting mitochondrial translation, tigecycline exhibits antitumor activity in acute myeloid leukemia (AML), glioblastoma, and retinoblastoma models (164–166). Doxycycline markedly reduces the expression of mitochondrially encoded proteins, thereby inhibiting cellular respiratory chain function (167). Previous studies have shown that doxycycline potentially inhibits cancer growth by inhibiting the propagation of cancer stem cells across diverse cell lines (168–171). A phase II clinical trial revealed that doxycycline significantly reduced the expression of the cancer stem cell marker CD44 in tumor tissue by ~40% in 8 of 9 patients (171).

However, mitochondrial translation inhibitors can also disrupt mitochondrial function in immune and stem cells, which limits their systemic tolerability. Most research is still at the preclinical or early clinical stage, highlighting the importance of optimizing dosing, developing tumor-targeted delivery strategies, and using biomarkers to guide patient selection.

### 5.3 Modulators of mitochondrial apoptosis

The mitochondrial pathway is governed by the balance between pro- and antiapoptotic members of the BCL-2 family that regulate MOMP and the subsequent release of cytochrome c (172). Pharmacological BH3 mimetics, such as venetoclax (a BCL-2-selective inhibitor) and navitoclax (a dual BCL-2/BCL-xL inhibitor), directly trigger MOMP and have achieved remarkable clinical efficacy in hematologic malignancies, including chronic lymphocytic leukemia and acute myeloid leukemia (173, 174). However, their success in solid tumors has been limited by the redundancy and compensatory expression of antiapoptotic proteins such as MCL-1 and BCL-xL, as well as metabolic rewiring that elevates the apoptotic threshold (175, 176).

Tumor cells frequently exploit oxidative phosphorylation and mitophagy to maintain mitochondrial fitness, thereby reducing BH3 mimetic sensitivity. Recent preclinical studies indicate that combining BH3 mimetics with agents targeting mitochondrial metabolism, autophagy, or mitophagy can restore apoptotic priming and overcome drug resistance (177). Additional strategies include the modulation of VDACs and the inhibition of the mitochondrial permeability transition pore (mPTP), both of which can increase mitochondrial permeability and sensitize tumor cells to apoptosis (178). Despite these advances, systemic inhibition of BCL-2 family proteins remains constrained by on-target toxicity, as normal hematopoietic and cardiac cells rely on BCL-xL and BCL-2 for survival, as illustrated by the dose-limiting thrombocytopenia associated with navitoclax (179). Consequently, rational combination therapy guided by functional BH3 profiling and metabolic biomarkers is emerging as a promising precision-medicine strategy to safely extend mitochondrial apoptosis modulation to solid tumors and resistant hematologic cancers (180).

### 5.4 Redox-directed therapies

Cancer cells typically maintain elevated basal ROS levels due to increased metabolic activity and increased mitochondrial function. However, further increases in ROS can overwhelm the antioxidant capacity of cancer cells, leading to oxidative damage and cell death. Prooxidant compounds exploit this vulnerability by selectively increasing oxidative stress within tumor cells. For example, elesclomol acts as a copper ionophore that transports copper ions into the mitochondria, where they catalyze redox cycling reactions and promote excessive mitochondrial ROS production to induce apoptosis in tumor cells (181). Similarly, piperlongumine, a natural alkaloid derived from Piper longum, elevates intracellular ROS and concurrently inhibits the prosurvival kinase IKKβ, resulting in cell cycle arrest and apoptosis across a variety of cancer cell types, including breast, prostate, and pancreatic cancer (182–184).

Moreover, therapeutic strategies that impair cellular antioxidant systems have gained increasing attention. Suppression of the glutathione peroxidase 4 (GPX4) enzyme or inhibition of the cystine/glutamate antiporter system xC^−^ disrupts cellular redox homeostasis, leading to uncontrolled lipid peroxidation and triggering ferroptosis—a distinct, iron-dependent, nonapoptotic mode of cell death (185). Small molecules such as RSL3, a GPX4 inhibitor, and emerging FSP1 inhibitors effectively induce ferroptosis in preclinical models by disabling key antioxidant defense mechanisms and increasing ROS accumulation (186).

Despite these promising strategies, redox-directed therapies are limited by a narrow therapeutic window, as excessive systemic oxidative stress can damage normal tissues. Hypoxia can increase ROS production through HIF-1α activation, affecting both tumor survival and the therapeutic response (187). To achieve controlled oxidative bursts within tumors, emerging approaches combine ROS inducers with mitochondrial antioxidants or metabolic modulators (188, 189). Nanoparticle-based delivery systems, which are responsive to tumor acidity or specifically targeted to mitochondria, have demonstrated improved spatial precision of ROS generation and increased cancer cell susceptibility while minimizing systemic toxicity (190).

### 5.5 Modulators of mitochondrial biogenesis and dynamics

Mitochondrial biogenesis and dynamics are governed by tightly coordinated processes of fission, fusion, and replication, which are regulated primarily by PGC-1α, TFAM, DRP1, and mitofusins (MFN1/2). In cancer, however, dysregulation of mitochondrial dynamics supports tumor plasticity, enabling cells to adapt to fluctuating energy demands and survive therapeutic stress. Excessive DRP1-mediated fission promotes the fragmentation of mitochondria and a glycolytic phenotype associated with chemoresistance. Pharmacologic inhibition of DRP1, such as mdivi-1, has been shown to suppress mitochondrial fission, reduce ATP generation, and trigger apoptosis in breast, lung, and colon cancer cells (191, 192). Conversely, promoting mitochondrial biogenesis through PGC-1α activation can restore oxidative metabolism and sensitize melanoma or prostate tumors to therapy, revealing the dual context-dependent role of these regulators (193, 194). TFAM, which controls mitochondrial DNA transcription and replication, and MFN2, which mediates mitochondrial fusion and organelle communication, have also emerged as modulators of cancer metabolism and signaling. Their manipulation can disrupt mitochondrial–nuclear crosstalk, alter reactive oxygen species signaling, and reprogram cellular fate (195–197). However, systemic manipulation of mitochondrial dynamics may affect normal tissues with high metabolic turnover, raising concerns about off-target effects. Moreover, many studies rely on cell lines, acute pharmacologic tools, or nonphysiologic dosing. Therefore, future studies combining *in vivo* pharmacology, live-cell imaging, single-cell transcriptomics, and spatial metabolomics could elucidate how dynamic mitochondrial behavior dictates therapeutic responsiveness.

## 6 Discussion

Mitochondrial function in eukaryotic cells reflects a dynamic interplay between eukaryotic and ancestral prokaryotic traits, yet this cooperative balance is disrupted during the transition to cancer. Such disruption reactivates metabolic and signaling programs reminiscent of prokaryotic states, in line with the Systemic-Evolutionary Theory of the Origin of Cancer (SETOC) and the concept of atavistic regression. Chronic microenvironmental stress—such as hypoxia, nutrient deprivation, and acidosis—perturbs the endosymbiotic coordination between the nuclear and mitochondrial subsystems, leading to phylogenetic reversion or “de-endosymbiosis,” whereby ancestral unicellular-like programs are awakened (198). This regression manifests metabolically as the classical Warburg effect, characterized by activated aerobic glycolysis, disrupted ETC, and a shift from OXPHOS to lactic acid fermentation, thereby conferring robustness, a proliferative advantage, and drug tolerance akin to that of primitive organisms. Concurrently, the TCA cycle becomes truncated, producing excessive reductive agents that fuel tumor growth, whereas abnormal glutamine metabolism intersects this altered cycle to supply precursors for lipid and nucleotide biosynthesis.

From a broader metabolic perspective, mitochondrial dysfunction cannot be viewed in isolation but rather as a central hub that coordinates the entire metabolic network of cancer cells. Impaired oxidative phosphorylation reprograms glycolytic flux, alters lipid and amino acid turnover, and promotes dependence on alternative substrates such as lactate, pyruvate, and fatty acids to sustain bioenergetic and biosynthetic demands. This systemic remodeling reflects the metabolic plasticity of cancer, whereby cells dynamically switch between glycolysis, glutaminolysis, and OXPHOS according to oxygen and nutrient availability. Gene mutations, mitochondrially related inflammation, and iron metabolism dysfunction further drive this prokaryotic transformation of mitochondrial metabolism, together shaping the remarkable adaptability and resilience of cancer cells and highlighting vulnerabilities as therapeutic targets.

The prokaryotic features of mitochondria provide an intriguing scope for cancer biology, offering novel therapeutic targets. Antibiotics that inhibit mitochondrial ribosomes and were initially designed against bacterial pathogens, such as doxycycline and tigecycline, impair mitochondrial translation and have shown efficacy against several cancers. In addition, mitochondria-specific drug delivery systems, such as lipophilic cations and mitochondria-targeting peptides, have the potential to increase drug selectivity and reduce systemic toxicity (199). Another prospective area is metabolic vulnerabilities associated with mitochondrial reprogramming. The truncated TCA cycle, the reliance of tumor mitochondria on glutamine metabolism, and the generation of oncometabolites such as succinate and 2-HG offer targets for metabolic therapies (200–202). Targeting mitochondrial inflammation and iron metabolism, such as by blocking mtDNA release (127) or restoring iron homeostasis (136), could reduce tumor-promoting microenvironmental factors and sensitize tumors to therapy. Moreover, mitochondrial transplantation and the development of artificial mitochondria have shown success in restoring cellular function in diseases associated with mitochondrial dysfunction (203). However, their applicability is currently limited primarily to conditions characterized by a deficiency of healthy mitochondria, and challenges such as inefficient mitochondrial delivery and integration of transplanted mitochondria into host cells further hinder their use in cancer treatment (204, 205).

Despite promising prospects, most mitochondria-targeted therapies remain in the *in vitro* or preclinical stage. One major challenge stems from the remarkable metabolic flexibility of cancer cells, which enables them to dynamically shift between glycolysis, glutaminolysis, and OXPHOS in response to environmental stress, oxygen fluctuations, and nutrient availability (206). This adaptability is supported by metabolic compensation mechanisms such as AMPK activation and HIF-1α signaling, which can attenuate apoptosis and increase cell survival under conditions of mitochondrial stress (207). Additionally, given that mitochondria are indispensable for ATP production in high-energy-demand tissues such as the heart and brain, the therapeutic window for mitochondria-targeted drugs is narrow. Targeting moieties such as triphenylphosphonium cations, while effective for mitochondrial accumulation, may disrupt the mitochondrial membrane potential in healthy tissues, causing off-target toxicity in neurons and cardiomyocytes (208). These toxicity risks underscore the need for tumor-selective delivery strategies. Metabolic heterogeneity across cancers further complicates therapy. Some tumors rely mainly on glycolysis, others on glutamine metabolism, and some on OXPHOS. The frequency and extent of disrupted mitochondrial activity, including diverse types of truncated TCA cycle functions, remain poorly defined across tumors. Considering these limitations, progress will require precision delivery systems, low-toxicity agents, and robust mitochondrial metabolic profiling to identify responsive patients. Ultimately, mitochondrial targeting may need to be integrated into a personalized metabolic therapy framework to overcome biological and clinical barriers.

In conclusion, the prokaryotic origins of mitochondria endow them with unique features that contribute significantly to cancer development. These insights help our understanding of tumorigenesis and inspire the potential of targeting mitochondrial dysfunction to develop novel and effective cancer therapies.
